# Harnessing the potential of pigeonpea and maize feedstock biochar for carbon sequestration, energy generation, and environmental sustainability

**DOI:** 10.1186/s40643-023-00719-3

**Published:** 2024-01-03

**Authors:** Nallagatla Vinod Kumar, Gajanan Sawargaonkar, C. Sudha Rani, Rajesh Pasumarthi, Santhosh Kale, T. Ram Prakash, S. Triveni, Ajay Singh, Moses Shyam Davala, Rohan Khopade, Rayapati Karthik, Bathula Venkatesh, Mandapelli Sharath Chandra

**Affiliations:** 1 Department of Agronomy, College of Agriculture, Professor Jayashankar Telangana State Agricultural University PJTSAU, Rajendranagar, Hyderabad 500030 India; 2 International Crops Research Institute for the Semi-Arid Tropics, Patancheru, Hyderabad 502324 India; 3 Agricultural Research Station, Jayashankar Telangana State Agricultural University PJTSAU, Vikarabad, Tanduru, Professor India; 4 AICRP On Weed Control, Professor Jayashankar Telangana State Agricultural University PJTSAU, Rajendranagar, Hyderabad500030 India; 5 Department of Agricultural Microbiology and Bio-Energy, College of Agriculture, Professor Jayashankar Telangana State Agricultural University PJTSAU, Rajendranagar, Hyderabad 500030 India; 6 IFS, Rajendranagar, PJTSAU, Hyderabad, 500030 India

**Keywords:** Slow pyrolysis, Carbon sequestration, Maize, Pigeonpea, Energetic retention efficiency, Reduction potential

## Abstract

Crop residues in agriculture pose disposal challenges and contribute to air pollution when burned. This study aims to use pigeonpea and maize stalks to produce biochar at different pyrolysis temperatures. Biochar can serve in carbon sequestration, as a soil amendment, and as an alternative fuel source. Pyrolysis was conducted at 400, 500, and 600 °C to examine the effects on physicochemical properties, fuel, and energy related properties. Increase in temperatures resulted in decrease of biochar yield, volatile matter, and O/C and H/C atomic ratios, while ash content and essential nutrients increased. Yield was observed to be higher in pigeonpea stalks derived biochar compared to maize stalks derived biochar at same pyrolysis temperatures. The yields of pigeonpea stalks derived biochar at 400 °C, 500 °C, and 600 °C are 34, 33 and 29%, respectively, and the yields of maize biomass-derived biochar at 400 °C, 500 °C, and 600 °C are 29, 28, and 26%, respectively. The organic carbon content is found to be higher in the biochar samples prepared at 600 °C, i.e., 10.44%, and 10.39% for pigeonpea and maize-derived biochar, respectively. The essential elements of biochar were increased with an increase in pyrolysis temperature except nitrogen which is conversely related to temperature. The biochar obtained through pyrolysis at 400 °C demonstrated superior characteristics compared to biochar produced at other temperatures. It exhibited a higher biochar yield, with approximately 84.60% for pigeonpea and 64.85% for maize fixed carbon content. Additionally, the energy retention efficiency was higher, reaching 67.33% for pigeonpea and 42.70% for maize-derived biochar at a pyrolysis temperature of 400 °C. The fixed carbon recovery efficiency was also notable at around 200.44% for PPS and 142.37% for maize biochar which is higher compared to biochar produced at other temperatures. Furthermore, the higher heating value (HHV) was approximately 30.75 MJ kg^−1^ for both the biochars, indicating their suitability as alternative solid fuels. A significant CO_2_ reduction potential of 84 CO_2_ eq kg^−1^ and 55 CO_2_ eq kg^−1^ was observed for pigeonpea and maize biochar, respectively. Hence, biochar is a promising and effective option for carbon sequestration, offering environmental benefits.

**Graphical Abstract:**

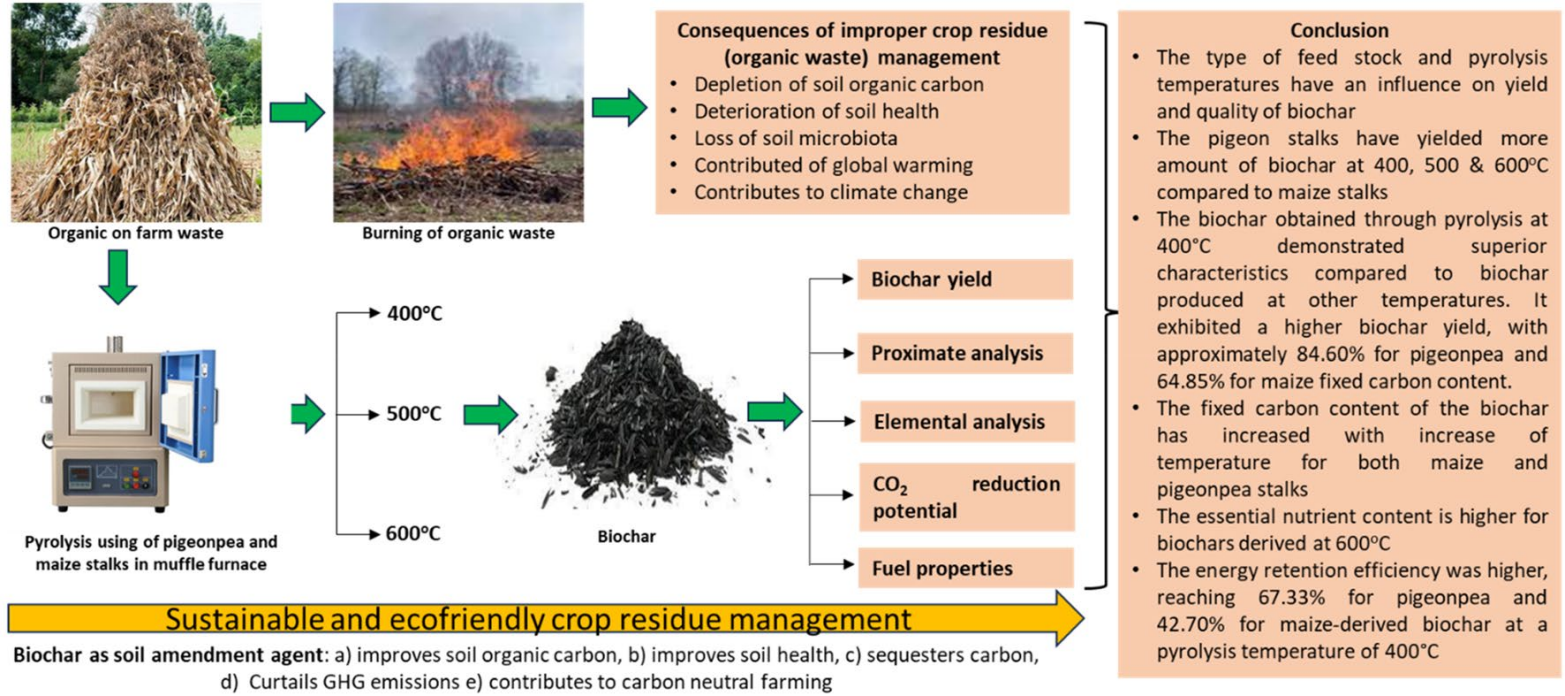

## Highlights

• Biochar produced from crop residues: a sustainable solution for decreasing atmospheric CO_2_ levels

•The temperature used during pyrolysis has a notable impact on the yield and characteristics of biochar. Biochar produced at 400°C shows superior characteristics, including higher CO_2_ reduction potential

•Biochar from both biomass sources meets the quality criteria for soil carbon sequestration

## Introduction

Biochar is a carbon-rich material produced through pyrolysis of biomass in low-oxygen conditions, which is cost-effective (Gul et al., 2015). pyrolysis typically occurs within the temperature ranges from 300 °C to 600 °C. In recent times, pyrolysis technology has gained popularity as an efficient way to use agricultural residues. This conversion process presents a novel opportunity to enhance the value of crop residues, offering various benefits including decreased bulkiness, reduced transportation costs, improved milling handling and storage (Masto et al. 2013). In line with the concept of the circular economy, the sustainable management of crop residues and biochar production can be optimized to minimize waste and resource inefficiencies. By adopting circular economy principles, we can create a more environmentally friendly and economically viable approach to biochar production and agricultural waste management (Yrjälä et al. 2022).

Biochar, a carbon-rich product derived from organic matter, offers a multitude of applications across various sectors due to its unique properties. It enhances soil health by improving nutrient retention, water-holding capacity, and microbial activity, while reducing reliance on chemical fertilizers. In animal farming, biochar serves as a feed additive to bolster animal health and mitigate methane emissions and is also beneficial in bedding materials for odour control and manure composting. Its role in anaerobic digestion is notable, where it increases surface area for microbial activity, boosting digestion efficiency and biogas production. Biochar's addition to compost piles can accelerate decomposition, improve compost quality, and diminish odours. In water treatment, its porous structure effectively adsorbs contaminants, aiding in purification processes (Osman et al. 2022).

Regarding soil remediation, biochar is instrumental in adsorbing pollutants, thereby reducing their environmental impact. In construction, it finds use in building materials like bricks or concrete, enhancing material properties and aiding in carbon sequestration. Its potential in energy storage is significant, particularly for developing improved electrodes for supercapacitors or batteries, thanks to its high conductivity and surface area (Osman et al. 2022). One of the most significant environmental benefits of biochar is its ability to sequester carbon (Kuttippurath et al. 2023).

Biochar has garnered recognition for its ability to decrease the H/C and O/C atomic ratios, as well as its elevated calorific value and energy density (Phanphanich and Mani 2011). The organic sources suitable for biochar production encompass forest residues, agricultural remnants (Prabha et al. 2015), and agro-industrial waste (Jothiprakash and Palaniappan 2014). In India alone, out of an estimated annual production of 511 million tonnes crop residue, around 92 million tonnes of crop wastes are incinerated each year (IARI 2012). Crop wastes are generated, with approximately 141 million tonnes considered surplus crop residue (Kaur 2017). The Government of India has estimated that farmers are producing annually around 18.53 and 19.73 million tonnes of pigeonpea and maize stalks, respectively (Government of India, Agricultural Statistics at a Glance 2018). Despite their high lignin content and limited digestibility, pigeonpea and maize stalks are seldom utilized as animal feed. Moreover, due to logistical hurdles and a lack of demand, only a fraction of these residues has been effectively utilized as an economical source of solid biomass fuel for cooking and heating purposes in rural areas, while a substantial portion remains overlooked within agricultural fields. Moreover, agricultural residues characterized by low bulk density and sluggish decomposition rates not only hinder soil preparation and crop establishment but also frequently result in their direct burning in open fields. This practice poses significant hazards to the environment, biodiversity, and human well-being (Venkatesh et al. 2018). Pyrolysis presents an opportunity for farmers to convert substantial quantities of crop leftovers into biochar, while incorporating agricultural residual biochar into the soil provides an efficient and appealing alternative to the harmful practice of open field burning (Venkatesh et al. 2018; Abhishek et al. 2021). However, the scalability of the pyrolysis technology and application of biochar as soil amendment depends on the availability of low-cost operational pyrolysis units or kilns at farmers’ fields and proper training to farmers.

Prior studies have revealed the substantial effect of feedstock and production conditions on various aspects of biochar, including yield (Keiluweit et al. 2010), physicochemical properties (Gaskin et al. 2008), total potential carbon (TPC), CO_2_ reduction potential (Allyson 2011), and energy properties (Lu et al. 2013). The potential ways of biochar usage can be figured out only by understanding the production process, characterization of feed stock materials and biochar produced from respective feed stock materials. For instance, biochar characterized by high recalcitrance can be utilized as a valuable material for carbon fixation. On the other hand, biochar with elevated elemental compositions or possessing high porosity holds the potential to significantly improve soil fertility when employed as amendment. These distinct attributes enable biochar to serve various purposes depending on the desired outcome, making it a versatile tool for enhancing agricultural practices and environmental sustainability (Alkharabshehet al. 2021). Additionally, biochar with higher heating values (HHV) can be employed in sol–gel production. According to a study by Gholizadeh & Hu (2021), biochar can remove up to 80% of heavy metals from contaminated soils, showcasing its potential in the field of pollutant removal. Biochar synthesized by pyrolyzing biomass, holds great chemical and biological stability, a high capacity for cation exchange, a large specific surface area, and a great concentration of functional groups (He et al. 2019). In a comprehensive review (Qian et al. 2023), the synergistic potential of biochar and compost in enhancing soil quality and crop yields was assessed across diverse soils, including tropical, temperate, dry, saline, and contaminated soils. The combined application of biochar and compost proved effective in mitigating various soil challenges, from mineralization rates to toxicity and contamination issues, highlighting its promise for sustainable soil improvement. However, there is currently limited information on how production conditions impact the composition quality and energy properties of biochar derived from pigeonpea stalks in India (Venkatesh et al. 2022a, b).

Conducting an in-depth investigation into the specific attributes of biomass material is crucial to understanding its pyrolysis behaviour and evaluating its viability as a biochar synthesis feedstock. Remarkably, no comprehensive research has been conducted thus far on the production and characterization of biochar from pigeonpea stalks. With the science of carbon sequestration progressing and a surplus of underutilized maize and pigeopea stalks readily available, the present study aims to investigate the influence of different pyrolysis temperatures biochar yield, physiochemical characteristics of biochar derived from maize and pigeonpea stalks.

## Material and methods

**Fig. 1:**
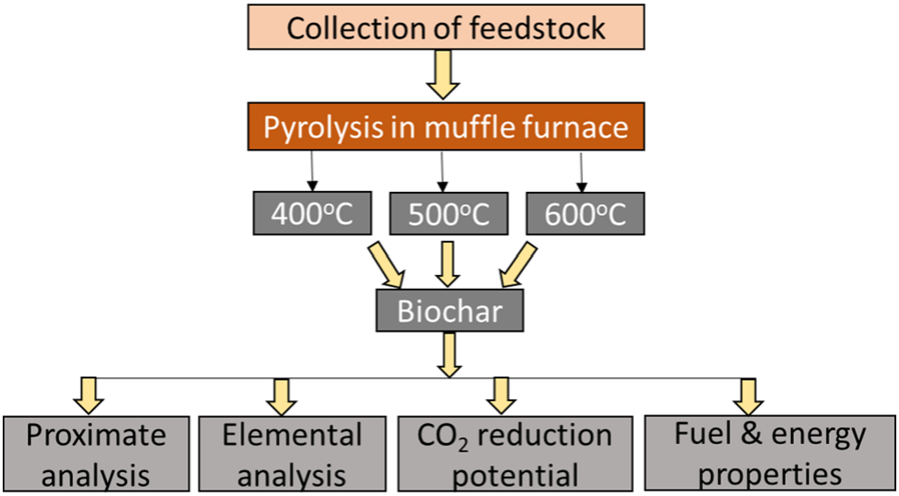
Schematic diagram illustrating the experimental steps

A step wise methodology of biochar production and characterization was represented in a schematic diagram (Fig. 1). Proximate analysis of biochar derived from maize biomass and pigeonpea stalks was conducted to estimate ash, moisture, volatile matter and fixed carbon content of the biochars and elemental analysis was carried out. H/C and O/C atomic ratios were estimated to determine the stability of biochar. CO_2_ reduction potential and fuel properties of biochar were characterized.

### Sample collection and preparation

Pigeonpea stalks and maize stalks were sourced from International Crop Research Institute for Semi-Arid Tropics (ICRISAT), Patancheru, Hyderabad, Telangana. The research station is located at an elevation of 461 m above mean sea level, positioned at 17° 15′ N latitude and 77° 35′ E longitude. The collected material was first cleaned to remove any impurities like soil or stones and then finely chopped into small pieces of 2–5 cm. Subsequently, they underwent an oven-drying process at 70 °C for a duration of 48 h. Afterward, the dried material was pulverized and passed through 20 mesh sieves, resulting in a particle size of 1 mm, which was suitable for muffle furnace biochar production.

### Biochar production and yield

The pyrolysis experiments were performed on pigeonpea stalks and maize residues in 11 L volume muffle furnace. A digital temperature regulator was placed on the furnace and pyrolysis took place in a controlled environment. The feedstocks were pyrolyzed at three different temperatures, i.e., 400, 500, and 600 °C. Prior research has demonstrated a noteworthy correlation between pyrolysis temperature and the yield and properties of biochar (Ghysels et al. 2020; Yang et al. 2020). These studies have provided evidence of the substantial influence that varying pyrolysis temperatures can have on the resulting biochar characteristics and quantity, so we have produced with three different pyrolysis temperatures. A continuous supply of nitrogen (99.9% purity) was maintained throughout the experiment to ensure an oxygen-deprived atmosphere (Ojha et al. 2020).

To ensure consistent pyrolysis, the materials were kept at a set temperature for an hour, while heating at a rate of 10 °C/min. For each pyrolysis cycle, approximately 500 g of feed stocks were utilized. The biochar samples were then cooled to room temperature in a nitrogen environment (Chen et al. 2020). Afterward, the biochar samples were subjected to a quenching process with distilled water to remove undesirable substances, specifically, organic molecules that have the potential to condense on the biochar during the cooling phase (Thomas 2021). They were then dried at 105 °C for 12 h [Li et al. 2020, b. The resulting biochar obtained from pigeonpea stalks was labelled as PPS400, PPS500, and PPS600, indicating biochar produced at temperatures of 400, 500, and 600 degrees Celsius, respectively. Similarly, biochar derived from maize stalks were designated as MB400, MB500, and MB600). All pyrolysis process at 400, 500, and 600 °C were performed in triplicate, and the average results were recorded and the triplicate samples of biochar underwent characterization to assess how the pyrolysis process influenced its proximate, elemental and fuel properties. The biochar yield (Qin et al. 2020)] was calculated using the following Eq. (1):

1 $$Biochar\, yield \left( \% \right) = \frac{Mass\, of\, biochar\, obtained\, \left( g \right)}{{Mass\, of\, feedstock\, loaded\, \left( g \right)}} \times 100.$$

### Proximate analysis

We conducted a proximate analysis following specific procedures for analysing cake and coal (ASTM D3172-13 2013). This analysis helped us to determine the volatile matter, moisture content, fixed carbon, and ash contents of both the initial feedstocks and the prepared biochar. We measured these values based on the weight of the samples on a dry basis after removing any moisture. The weight that remained after accounting for moisture content, volatile matter and ash contents was considered as fixed carbon content (FC):

2 $${\text{Moisture }}\left( \% \right) = { }\frac{A - B}{B} \times 100,$$

3 $${\text{Volatile matter }} = { }\frac{B - C}{B} \times 100,$$

4 $${\text{Ash content }}\left( \% \right) = { }\frac{C - D}{C} \times 100,$$

where A: mass of the air-dried sample in grams, B: mass of the sample after being dried at 105 °C for 2 h in grams, C: mass of the sample after being dried at 950 °C for 6 min in grams, and D: mass of the residue remaining after drying at 750 °C for 6 h in grams.

5 $${\text{Fixed carbon }}\left( \% \right) = { }100 - \left( {{\text{moisture }} + {\text{ volatile matter }} + {\text{ ash}}} \right).$$

### Elemental analysis

We conducted elemental analysis of both biomass samples and prepared biochar at different temperatures. Carbon (C) and nitrogen (N) measurements were obtained as per the protocol mentioned by Venkatesh et al., (2013a, b), using a CHNS analyzer (Elementar, model: UNICUBE) having a detection range of 0.50–20 mg for C, 0.05–5 mg for N, 0.01–1 mg. For the extraction of phosphorus (P), potassium (K), sulphur (S), boron (B), zinc (Zn), copper (Cu), iron (Fe), and manganese (Mn), specific methods were followed. Sodium bicarbonate was used for P extraction based on the Olsen and Sommers (1982), ammonium acetate for K extraction following the Helmke and Sparks (1996), 0.15% calcium chloride for S extraction following Tabatabai et al. (1996), hot water for B extraction based on Keren et al. (1996), and diethylene triamine penta-acetic acid (DTPA) reagent for Zn, Cu, Fe, and Mn extraction as per the Lindsay and Norvell method (1978). The colorimetric approach was employed to estimate P levels as per Olsen et al., (1954), while the atomic absorption spectrophotometer (AAS-Agilent-Savant AA) was utilized to measure K. The analysis of S, B, Zn, Cu, Fe, and Mn was conducted using inductively coupled plasma atomic emission spectroscopy (ICP-AES: Agilent 5110ICP-OES) as per the protocol mentioned by Onorevoli et al., (2018), at the Charles Renard Analytical Laboratory (CRAL) in the ICRISAT.

### Biochar stability

The H/C and O/C atomic ratios obtained through proximate analysis can be useful in characterizing biochar, they should be considered as part of a broader analysis of stability. These atomic ratios were calculated using Eqs. (6) and (7) by Klasson 2017. The equations are as follows:

6 $$\frac{{\text{H}}}{{\text{C}}} = { }0.397{ } \times { }\left( {\frac{{{\text{VM}}}}{{{\text{FC}}}}} \right) + { }0.251,$$

7 $$\frac{{\text{O}}}{{\text{C}}} = 0.188 \times \left( {\frac{VM}{{FC}}} \right) + 0.035.$$

In this context, we utilize the following notation:

FC: represents percentage of fixed carbon present in biochar, VM: denotes percentage of volatile matter contained within biochar, H represents hydrogen content in biochar sample, C represents carbon content in biochar sample, and O represents oxygen content in biochar sample.

It is important to note that the equations assume complete combustion of the biochar sample during proximate analysis. This may not account for all forms of carbon, hydrogen, and oxygen present in biochar structure.

### Carbon dioxide reduction potential

The carbon dioxide reduction potential is determined using Tesfamichael et al. (2018) methodology. The first step is to calculate the total potential carbon (TPC) following Eq. 8:

8 $$TPC \,in \, biochar\, \left( {g\,of C kg^{ - 1} of biochar} \right)\, = \,Total\, biochar \,yield \,\left( {kg \,of\, biochar \,kg^{ - 1} \, of \,stalk} \right)\, \times \, Fixed\,carbon \,\left( {kg\, of\, FC\, kg^{ - 1} \,of\, biochar} \right).$$

Afterwards the CO_2_ reduction potential (CO_2_ eq kg^−1^ of biochar) was determined using Eq. 9 as follows:

9 $$\begin{gathered} {\text{Co}}_{2} {\text{ reduction potential total}} = {\text{ potential carbon }}\left( {{\text{TPC}}} \right){\text{ of biochar }}\left( {{\text{g of C }}kg^{ - 1} {\text{of biochar}}} \right)\left( {\frac{80}{{100}}} \right)\left( {\frac{44}{{12}}} \right). \hfill \\ \hfill \\ \end{gathered}$$

### Fuel properties

The empirical method developed by Parik et al. (2007) was employed to calculate hydrogen (H) and oxygen (O) elemental composition of the pigeonpea stalks (PPS) and maize stalks (MB). Equations 10 and 11 were used for these calculations and were based on the findings obtained from the proximate analysis conducted on the samples:

10 $${\text{H }}\left( {\text{\% }} \right) = { }0.052{ } \times {\text{ FC }} + { }0.062{ } \times {\text{ VM,}}$$

11 $${\text{O}}\,\left( {\text{\% }} \right)\, = { }\,0.304{ } \times { }\,{\text{FC }}\, + \,{ }0.476\,{ } \times { }\,{\text{VM}}{.}$$

The next step is to estimate calorific or higher heating value (HHV) in both samples and prepared biochar (from PPS and MB) using Eq. 12:

12 $${\text{HHV }}\left( {{\text{Mj kg}}^{ - 1} } \right) = { }0.3536{ } \times {\text{ FC }} + { }0.1559{ } \times {\text{ VM }}{-}{ }0.0078{ } \times {\text{ ash}}{.}$$

The energy densification (Ed), HHV improvement (HHVi), FC densification (FCd), FC recovery efficiency (FCre), fuel ratio (Fr) and energetic retention efficiency (ERE), of the biochar were determined by employing specific equations. These calculations were based on the yield and proximate analysis data associated with the biochar (Kim et al. 2016; Nakason et al. 2019). These parameters were calculated using the equations below:

13 $${\text{Energy }}\,{\text{densification }}\,\left( {{\text{Ed}}} \right) = \,\frac{{\text{ HHV of dried biochar}}}{{\text{HHVof dried sample }}},$$

14 $${\text{Energy }}\,{\text{retention}}\,{\text{efficiency }}\left( {{\text{ERE}}} \right)\left( {\text{\% }} \right)\, = \,{\text{Ed }}\, \times \,{\text{biochar yield,}}$$

15 $${\text{HHV improvement }}\left( {\text{HHVi }} \right) = \frac{{{\text{HHV of dried biochar }} - {\text{ HHV of dried sample}}}}{{\text{HHV of dried sample}}},$$

16 $${\text{Fixed carbon densification }}\left( {{\text{FCd}}} \right) = \,\frac{{\text{FC of dried biochar}}}{{\text{FC of dried biochar}}}{, }$$

17 $${\text{Fixed carbon recovery efficiency }}\left( {{\text{FCre}}} \right)\left( {\text{\% }} \right) = {\text{ FCd }} \times {\text{ yield of biochar , }}$$

18 $${\text{Fuel ratio }}\left( {{\text{Fr}}} \right) = { }\frac{{\text{Fixed carbon in biochar}}}{{\text{ Volatilematter in biochar }}}{ }{\text{.}}$$

### Statistical analysis

The data are reported as the mean ± standard error mean (*n* = 3). In order to assess significant variations between the treatments, paired t was performed, using a *p*-value of 0.05 as the critical threshold for determining statistical significance.

## Results and discussion

### Biochar yield

The biochar was produced from pigeonpea and maize stalks at three different pyrolysis temperatures: 400 °C, 500 °C, and 600 °C. The biochar yields from pigeonpea stalks were 34%, 33%, and 29%, respectively (Table 1). The biochar yields of maize stalks were 29%, 28%, and 26% at the corresponding pyrolysis temperatures. The yields of biochar prepared from maize stalks are slightly lower than pigeonpea stalks. Based on the findings, it was observed that the production of biochar from both pigeonpea stalks and maize stalks decreased as the pyrolysis temperature increased. This decline in biochar yield can be linked to factors such as differences in feedstock composition, pyrolysis conditions, and the inherent properties of the pigeonpea stalks and maize stalks. The temperature at which pyrolysis occurs is important for breaking down organic material and creating biochar. When the temperature is higher, there is more thermal decomposition and volatile compounds are released more quickly. This can result in lower biochar production (Zhang et al. 2017). The moisture content of biomass affects the production of biochar. Higher moisture levels require more energy input, result in higher biomass consumption, and ultimately lead to lower biochar yield. To achieve optimal biochar production, it is recommended to use biomass with a lower mass fraction of moisture, typically less than 11% (Khuenkaeo and Tippayawong 2020; Canal et al. 2020). This choice ensures efficient utilization of resources and maximizes the biochar yield, making it a preferred approach in biochar production. As both the raw materials biomasses (maize and pigeonpea) have less than 11% moisture content, both are suitable feedstocks for biochar production. These findings align with previous studies that have investigated the impact of pyrolysis temperature on biochar yield from various feedstocks. For instance, Yaashikaaet al. (2020) reported similar trends in biochar yield reduction with increasing pyrolysis temperature for different biomass sources. Additionally, Chen et al. (2014) investigated biochar production from different agricultural residues and found that higher pyrolysis temperatures led to decreased biochar yields. Numerous studies have shown that biochar yields increase with higher pyrolysis temperatures (Titiladunayo et al. 2012; Brewer et al. 2014; Cao et al. 2018; Hernandez-Mena et al. 2014). Similarly, Sahoo et al. (2021) found that when pigeonpea stalks are pyrolyzed, the biochar yield decreases with an increase in temperature from 400 °C to 600 °C, a finding consistent with our observations.

**Table 1:** The proximate analysis of pigeonpea stalk and maize stalks

Sample	Moisture content (wt %, wb)	Fixed carbon (wt %. Db)	Volatile matter (wt %. Db)	Ash (wt %. Db)	Biochar yield (%)
Pigeonpea	7.54 ± 0.14	14.35 ± 1.04	76.64 ± 5.58	2.00 ± 0.15	–
Maize	8.11 ± 0.01	13.21 ± 0.84	73.56 ± 4.68	1.79 ± 0.12	–
PPB 400	6.32 ± 0.12	84.60 ± 7.58	5.50 ± 0.49	3.12 ± 0.28	34.00 ± 2.47
PPB 500	6.18 ± 0.62	85.21 ± 5.95	4.70 ± 0.33	4.35 ± 0.30	33.00 ± 2.10
PPB 600	6.10 ± 0.07	86.00 ± 2.51	3.90 ± 0.12	4.99 ± 0.14	29.00 ± 2.60
MB 400	7.35 ± 0.16	64.85 ± 4.58	6.00 ± 0.42	18.56 ± 1.31	29.00 ± 2.03
MB 500	7.21 ± 0.22	65.00 ± 4.06	5.30 ± 0.33	23.44 ± 1.46	28.00 ± 0.82
MB 600	7.14 ± 0.70	67.56 ± 2.49	2.90 ± 0.11	28.12 ± 1.03	26.00 ± 1.84

Values is the mean ± standard error of mean

*PPB* pigeonpea biochar, *MB* maize biochar, *wb* wet basis, *Db* dry basis

The study emphasizes the significance of pyrolysis optimization to attain biochar yield and maximize biomass utilization. It is important to consider biochar quality, stability, and its impact on soil health and carbon sequestration when analysing its potential. The proximate analysis is presented in the following section.

### Proximate analysis:

The proximate analysis of feedstock and biochar prepared from maize and pigeonpea stalks is presented in Table 1. Volatile matter, moisture content, ash and fixed carbon for biomass and prepared biochar were analysed at three different pyrolysis temperatures, i.e., 400 °C, 500 °C and 600 °C. Since the proximate analysis is a method of assessing recalcitrance, the evaluation of biochar is significantly influenced by temperature (Singh et al. 2020). The pyrolysis conditions at 600 °C across both pigeonpea biochar (PPB) and maize biochar (MB) produced the lowest volatile matter, with MB having the lowest volatile matter about 2.90%, at pyrolysis temperature of 600 °C. Generally, as the pyrolysis temperature rises from 400 °C to 600 °C for biochar made from various biomasses, there's a reduction in volatile matter. This decrease can be attributed to incomplete carbonization and the existence of functional groups like C=O and C=H, as noted by Muigai et al. in 2021. The highest volatile matter percentage was recorded at MB 400 about 6.0%, while the lowest was recorded at MB 600 at 2.90%. Moreover, Li et al. (2018) also reported that there is a negative correlation between volatile matter and pyrolysis temperature, which is also reflected in our study. The ash content increases as the pyrolysis temperature increases, with the highest ash content being 28.12% at 600 °C for MB feedstock. When comparing ash content in different feedstocks with the same pyrolysis temperature, MB feedstock has a greater ash concentration than PPB feedstock, with a difference of roughly 15% at 400 °C. As the pyrolysis temperature increases, the disparity in ash content between the MB and PPB feedstocks becomes more pronounced. At 600 °C, this difference reaches approximately 25% owing to the thermal decomposition of the labile fraction, which enhances the proportion of minerals present. Additionally, higher pyrolysis temperatures lead to the release of carbon as volatile compounds. This observation has been discussed in studies conducted by Ghidotti et al. (2017) and Sun et al. (2017). The content of volatile matter and ash in biochar symbolizes two different carbon reservoirs: the labile pool and the non-labile pool, as explained by Lehmann and Joseph (2009a, b). This understanding is in line with the study carried out by Titiladunayo et al. (2012), exploring the pyrolysis of lignocellulosic wastes at a range of temperatures from 400 to 800 °C. Their research demonstrated a decline in volatile matter as the pyrolysis temperature increased, which was ascribed to mineral enrichment, aromatization, and the decomposition of lignocellulosic constituents. The findings are consistent with those presented by Domingues et al. (2017). The ash content identified in biochar derived from wood was discovered to be below 7%, a result that resonates with the studies of Mukome et al. (2013) and Wang et al. (2013).

Fixed carbon refers to the non-labile carbon fraction that persists after thermal degradation (Lehmann and Joseph, 2009a, b) and is mainly impacted by the type of the feedstock. In both feedstock instances, a notable increase in the content of fixed carbon was observed as the pyrolysis temperature rose (Zhao et al. 2014). Specifically, the percentage of fixed carbon increased with temperature elevation, resulting in a difference of 1.4% between PPB 400 and PPB 600, as well as a difference of 2.71% between MB 400 and MB 600. These findings highlight the impact of pyrolysis temperature on the fixed carbon content of biochar derived from the respective feedstocks. The pyrolysis process seems to have a greater effect on fixed carbon percentage for the MB type compared to the PPB type, with a difference of 2.71% between MB 400 and MB 600, and a difference of only 1.4% between PPB 400 and PPB 600. The fixed carbon percentage values of PPB 600 and MB 600 are relatively high compared to the others, which could suggest that the pyrolysis process at that temperature had a positive impact on the outcome of the experiment. The volatile content of pyrolyzed carbon (PyC) corresponds to its labile component, while a lower volatile matter content, accompanied by higher fixed carbon, is indicative of increased stability and resistance to decomposition. This relationship suggests that biochar with higher fixed carbon content possesses greater stability or durability, as it contains a smaller proportion of labile components that are prone to decomposition (Singh et al. 2020). Therefore, biochar prepared from pigeonpea stalks is more stable or resistant to decomposition as compared to biochar prepared from maize stalks. The ash content is also lower in pigeonpea-derived biochar as compared maize stalks derived biochar. Additionally, biochar with reduced ash content is more convenient for transportation and application in the soil as they are less prone to windblown loss (Tomczyk et al. 2020). Furthermore, pigeonpea biochar had a higher carbon content compared to maize biochar at the same pyrolysis temperature. The reason for this is the greater amount of cellulose and lignin present in the pigeonpea stalks, coupled with a reduced fraction of hemicellulose compared to maize stalks. Such observations are in alignment with the studies conducted by Roy et al. (2019) and Ghysels et al. (2020). The rise in the carbon mass fraction is due to intensified polymerization reactions that result in more condensed carbon formations and the elimination of surface functional groups (–OH) during the dehydration process of biochar creation (Mukome et al. 2013).

### Nutrients composition

**Fig. 2:**
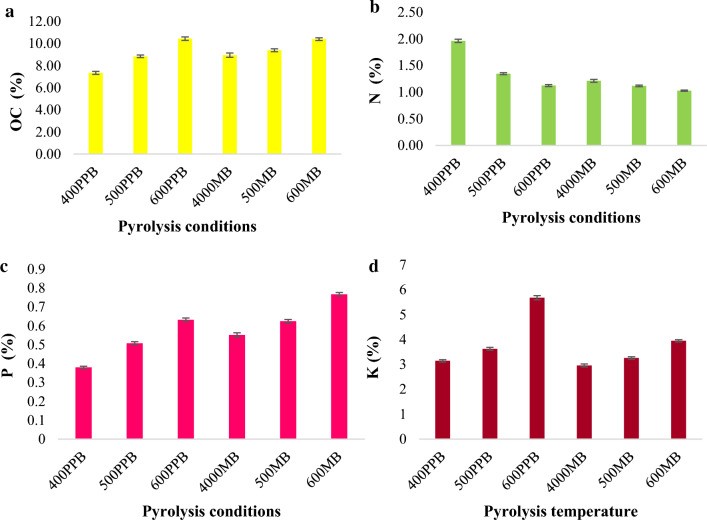
Effect of pyrolysis temperatures on OC (**a**), N (**b**), P (**c**) and K (**d**) nutrient content of pigeonpea and maize-derived biochar

The highest organic carbon (OC) value was observed at 600 °C pyrolysis condition in pigeonpea-derived biochar followed closely by MB 600, indicating that these conditions were the most effective in terms of OC content (Fig. 2). PPB conditions (400, 500) had lower oxidative conversion values than MB conditions (400, 500), suggesting that the type of biomass (PPB or MB) used in the pyrolysis process could affect the efficiency of the reaction. Although the organic carbon values were generally higher for MB conditions, MB 400 had a slightly higher value (8.94–9.08%) than PPB 400 (8.84–8.97%), which could indicate a more favourable reaction under certain circumstances or conditions. The depolymerization of organic matter during pyrolysis leads to the release of volatile gases and the production of biochar, which is rich in carbon. The volatile gases include compounds such as water, carbon dioxide, methane, and other organic compounds. The loss of these volatile compounds leaves behind a higher concentration of carbon in the biochar, resulting in an increased organic carbon content. These finding suggests that higher pyrolysis temperatures promote the production of biochar with increased organic carbon content, which is consistent with previous studies (Uzoma et al. 2011). In contrast, the nitrogen content in both pigeonpea and maize biochar exhibited a decreasing trend as the pyrolysis temperature increased. However, notable nitrogen concentrations were observed at 400 °C, with Pigeonpea biochar is containing 1.96–1.99% nitrogen and maize biochar is containing 1.13–1.14% nitrogen. At higher pyrolysis temperature, the organic nitrogen compounds undergo thermal decomposition and volatilization, leading to a reduction in nitrogen concentration in the resulting biochar (Fig. 2b). This finding indicates that lower pyrolysis temperatures can help preserve nitrogen content in biochar, which aligns with similar observations by Uzoma et al. (2011).

**Fig. 3:**
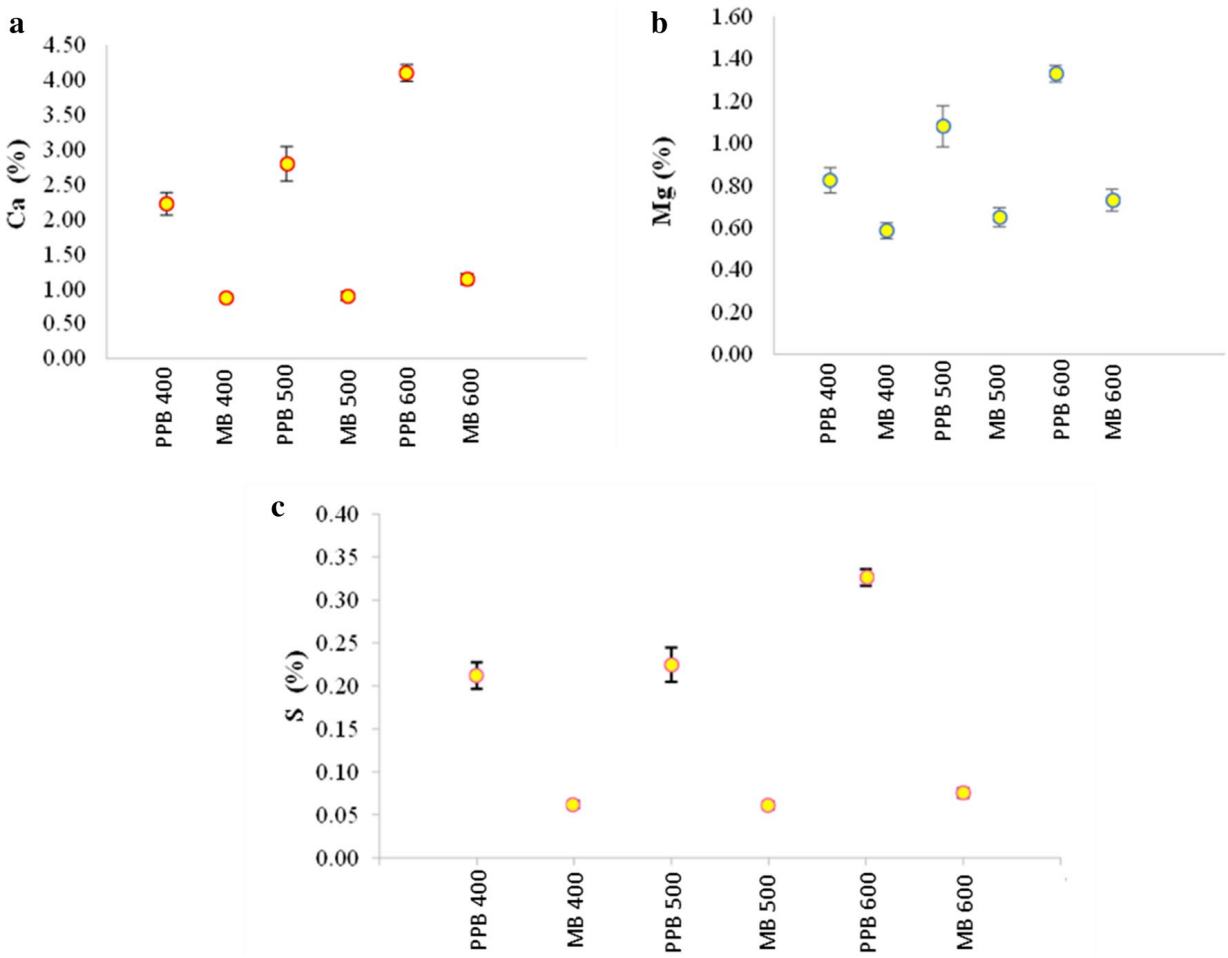
Effect of pyrolysis temperatures on Ca (**a**), Mg (**b**) and S (**c**) content of pigeonpea and maize-derived biochar

**Fig. 4:**
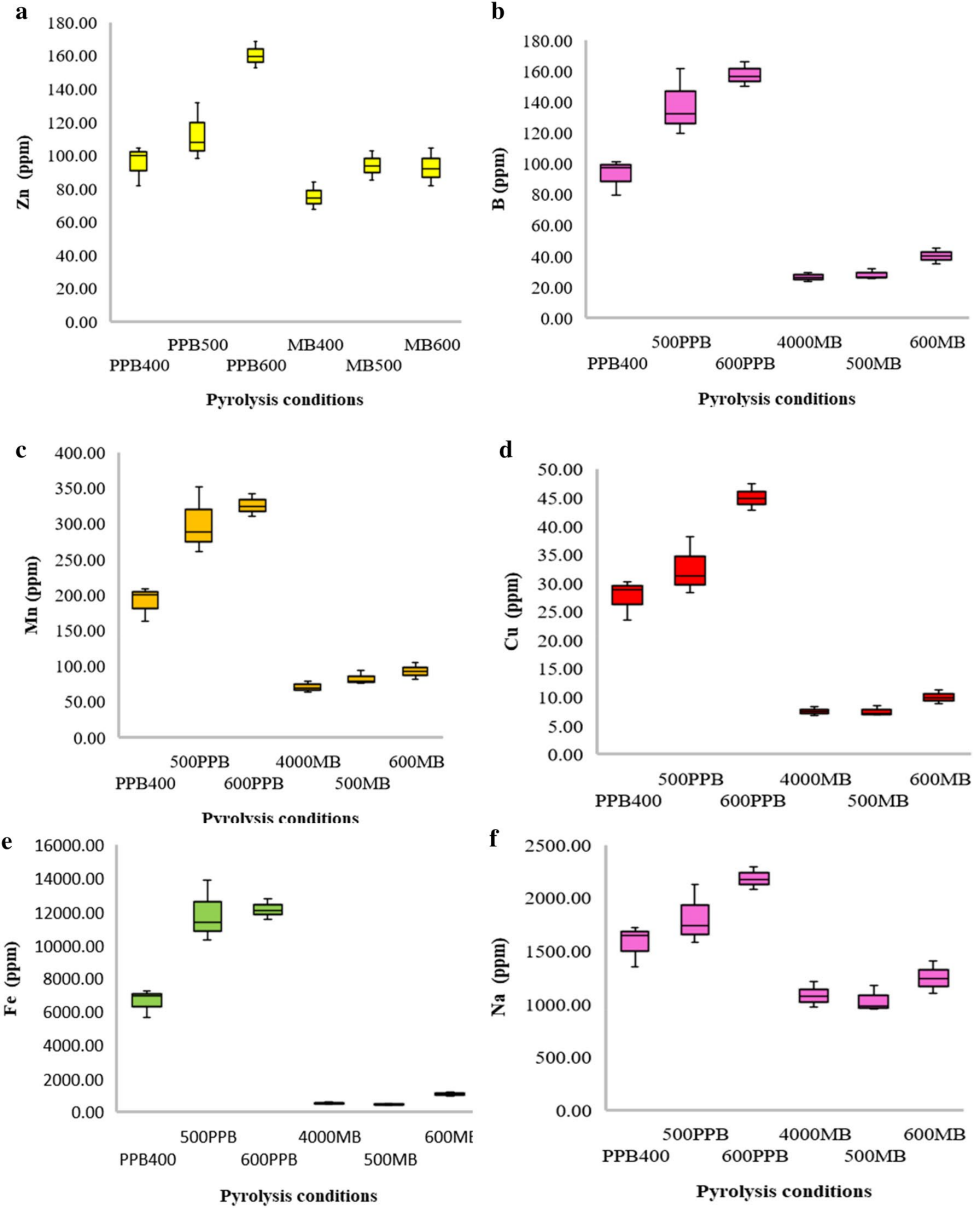
Effect of conditions on Zn (**a**), B (**b**), Fe (**c**), Cu (**d**), Mn (**e**), Na (**f**) content of pigeonpea and maize

Additionally, the concentrations of essential nutrients such as phosphorus (P), potassium (K), calcium (Ca), magnesium (Mg), sulphur (S) and various micronutrients (Zn, B, Fe, Cu, Mn and Na) were analysed in both maize and pigeonpea biochar. The results showed an upward trend in nutrient content with increasing pyrolysis temperatures (as shown in Figs. 3, 4) for both types of biochar. These elements are less susceptible to volatilization during pyrolysis due to their resistance to heating. This trend is in line with the findings reported by Chellappan et al. (2018) and supports the notion that nutrient concentrations in biochar can be influenced by pyrolysis temperature.

The observed results can be attributed to initial nutrient concentrations present in the feedstock materials, as well as the reduced losses of these nutrients during volatilization. Uzoma et al. (2011) also emphasize the role of initial nutrient content and reduced volatilization losses in nutrient retention within biochar. The findings of the current study contribute to the existing knowledge on nutrient dynamics in biochar and are consistent with previous research.

To bolster these findings, more studies have been conducted to examine the effect of pyrolysis temperature on the nutrient composition of biochar. For instance, Liu et al. (2013a, b) examined the effect of pyrolysis temperature on nutrient retention in biochar produced from crop residues and discovered that higher temperatures resulted in elevated concentrations of P, K, Ca, and Mg, which is in agreement with the findings of the present study.

### Biochar stability

The hydrogen (H), oxygen (O), hydrogen-to-carbon (H/C) ratio, and oxygen-to-carbon (O/C) ratio of biochar obtained from maize and pigeonpea stalks were analysed across various pyrolysis temperatures (Table 2). The results revealed variations in the elemental composition and ratios between the two biochar, indicating differences in their chemical characteristics. These variations can be attributed to several factors related to the feedstock composition and pyrolysis conditions. These factors play a significant role in shaping the elemental composition and characteristics of biochar.

**Table 2:** Estimates of H, O, O/C and H/C atomic ratios of pigeonpea and maize biochar

	Hydrogen	Oxygen	H/C	O/C
PPB 400	4.74 ± 0.34	28.34 ± 2.06	0.07	0.39
PPB 500	4.72 ± 0.30	28.14 ± 1.79	0.06	0.37
PPB 600	4.71 ± 0.42	28.00 ± 2.50	0.06	0.35
MB 400	3.74 ± 0.26	22.57 ± 1.57	0.05	0.30
MB 500	3.71 ± 0.11	22.28 ± 0.65	0.04	0.27
MB 600	3.69 ± 0.26	21.92 ± 1.54	0.04	0.25

Values are the mean ± standard error of mean

The increased hydrogen content noted in biochar derived from pigeonpea is due to the greater presence of hydrogen-rich compounds in pigeonpea stalk, including cellulose and hemicellulose. These compounds have a higher hydrogen-to-carbon ratio, leading to increased hydrogen content in the resulting biochar (Yang et al. 2016). On the other hand, maize stalks may contain fewer hydrogen-rich compounds, resulting in lower hydrogen content in the derived biochar. The significance of hydrogen content lies in the potential reactivity and reducing properties of biochar. Higher hydrogen content can enhance the reducing capacity of biochar, making it suitable for applications such as soil amendment and pollutant remediation (Budai et al. 2021). The higher oxygen content observed in pigeonpea stalk-derived biochar can be attributed to the presence of oxygen-rich functional groups, including carboxyl and hydroxyl groups, in the feedstock. These functional groups contribute to the oxygen content in the resulting biochar (Yang et al. 2016). Conversely, maize stalks may contain fewer oxygen-rich compounds, resulting in lower oxygen content in the derived biochar. The oxygen content of biochar is significant as it influences its stability, surface reactivity, and potential for carbon sequestration. Oxygen-containing functional groups can contribute to biochar’s sorption capacity and its ability to interact with pollutants in the environment (Cao et al. 2018).

The decrease in hydrogen and oxygen content in biochar with increasing pyrolysis temperature can be attributed to the thermal decomposition and volatilization of functional groups containing hydrogen and oxygen during the pyrolysis process. As the temperature rises, the thermal degradation reactions become more prominent, leading to the release of volatile components. The study’s findings align with previous research, including Chen et al. (2014), Zhang et al. (2019), and Budai et al. (2021), which also found that pyrolysis temperature increased the breakdown of hydrogen and oxygen content. In terms of hydrogen content, the study found that pigeonpea biochar had a higher H content compared to maize biochar. The reason for this difference could be because of the natural composition and structure of the materials used. Pigeonpea biochar has more hydrogen content because of its higher lignin content, which usually has more hydrogen than the cellulose or hemicellulose found in pigeonpea stalks. Regarding oxygen content, the results showed that pigeonpea biochar exhibited a higher O content compared to maize biochar. This difference could be attributed to the higher cellulose and hemicellulose content in pigeonpea stalks, which are known to have higher oxygen content than lignin.

The H/C ratio of biochar created from maize stalks was lower than that made from pigeonpea stalks. This indicates that the biochar from maize had a more stable and aromatic structure. Both biochar samples had generally low H/C ratios, indicating that they were highly carbonized and had undergone significant aromatization during the pyrolysis process, surpassing the original biomass materials. It is important to note that the biochar produced from both types of biomass met the quality standards set by the European Biochar Certificate and the International Biochar Initiative, with an H/C ratio below 0.7 (Schmidt et al. 2015). This indicates that they are suitable for soil carbon sequestration, which is an important part of sustainable agricultural practices. Among the biochar samples, the one made from pigeonpea stalk at 400 °C had the highest H/C ratio of 0.11, meaning it had a higher content of organic material compared to the others. The decrease in the O/C ratio across all biochar samples indicated a reduced hydrophilicity, which means oxygen-containing functional groups were removed and a dehydration process occurred during pyrolysis (Roy et al 2019). This change caused a drop in both the H/C and O/C ratios, which increased the stability and carbon-rich nature of the biochar.

The decrease in the H/C atomic ratio observed in both pigeonpea and maize biochar can be attributed to increased aromatization, which suggests improved structural stability (Kaewtrakulchai et al. 2018). The decrease in the atomic ratio of hydrogen-to-carbon (H/C) is caused by dehydration and dehydrogenation actions, along with the fragmentation and breaking of weak hydrogen bonds, according to the observations made by Qian et al. (2013) during the conversion of biochar. A lower O/C atomic ratio signifies that more carbonization has occurred due to the removal of oxygen through dehydration and decarboxylation processes (Shen et al 2017). These results demonstrate the advantageous characteristics of biochar, such as its structural stability and resistance to degradation in soil environments. The study conducted by Wei et al. (2017) revealed that the higher pyrolysis temperatures led to the production of biochar with increased aromaticity and decreased H/C and O/C atomic ratios compared to biochar produced at lower temperatures. Our findings are in line with this pattern, as all the pyrolysis experiments conducted with pigeonpea and maize resulted in biochar exhibiting decreasing H/C and O/C atomic ratios as the temperature was raised. This suggests an improvement in fuel characteristics comparable to lignite, sub-bituminous, and bituminous coal, leading to higher heating values.

The lower O/C ratio in both types of biochar at all pyrolysis temperatures can be attributed to the removal of oxygen-containing functional groups, such as carboxyl and hydroxyl groups, during the pyrolysis process. High-temperature pyrolysis promotes the release of volatile oxygen-containing compounds, resulting in a decrease in the O/C ratio (Sun et al., 2020). The O/C ratio is significant as it reflects the carbon stability and carbon sequestration potential of biochar. Biochar with lower O/C ratios are expected to exhibit higher stability and resistance. The biochar derived from maize and pigeonpea residues in this study, at various pyrolysis temperatures, fulfilled the crucial quality requirements of H/C < 0.7 and O/C < 0.4 for effective soil carbon sequestration, as stipulated by the European Biochar Certificate and the International Biochar Initiative. These criteria ensure that the biochar is suitable for effectively capturing and storing carbon in the soil, aligning with the goals of sustainable land management practices (Venkatesh et al 2022a, b).

### CO_2_ reduction potential

The study provides insights into biochar's CO_2_ reduction potential from maize and pigeonpea biomass at various pyrolysis temperatures. We examined three pyrolysis temperatures, namely 400 °C, 500 °C, and 600 °C, and evaluated the total potential carbon (TPC) and CO_2_ reduction potential of the resulting biochar (Table 3). Our findings indicate that as the pyrolysis temperature increased from 400 °C to 600 °C, both pigeonpea and maize biochar exhibited a decrease in TPC. This decrease can be attributed to fact that higher pyrolysis temperatures result in lower production of polycyclic aromatic hydrocarbons. Additionally, higher temperatures often lead to a more complete combustion of organic matter, resulting in reduced aromatic hydrocarbon content in the biochar.

**Table 3:** Estimates of total potential carbon (TPC) and CO_2_ reduction potential in biochar of pigeonpea and maize biochar

Pyrolysis conditions	TPC in biochar (g of C kg^−1^ of biochar)	CO_2_ reduction potential (CO_2_ eq kg^−1^)
PPB 400	287.64	84.22
PPB 500	281.19	82.33
PPB 600	249.40	73.02
MB 400	188.07	55.07
MB 500	182.00	53.29
MB 600	175.66	51.43

The biochar produced at 400 °C demonstrated the highest TPC values for both pigeonpea and maize biochar (Table 3). The TPC values were found to be 287.64 g kg^−1^ for pigeonpea biochar and 188.07 g kg^−1^ for maize biochar. Similarly, the CO_2_ reduction potential was highest for biochar produced at 400 °C, with values of 842.21 CO_2_ eq kg^−1^ for pigeonpea biochar and 55.07CO_2_ eq kg^−1^ for maize biochar.

The conversion of maize and pigeonpea stalks into biochar through pyrolysis offers several benefits. First and foremost, biochar is recognized as a highly stable form of carbon that exhibits remarkable resistance to microbial degradation. The exceptional stability of biochar enables it to endure in the soil for an extended duration, leading to effective sequestration of atmospheric CO_2_ and a reduction in carbon dioxide emissions. The significant amounts of organic carbon found in biochar derived from pigeonpea and maize at 400 °C further reinforce their potential as long-term carbon storage solutions within the soil. By converting atmospheric carbon dioxide into a stable carbon form within biochar, this process actively contributes to mitigating climate change by reducing greenhouse gas emissions. By preventing the rapid mineralization of carbon present in crop waste through biological decomposition or in situ burning, biochar production offers an effective strategy for carbon sequestration (Guo and Chen 2014).

Our study's findings are consistent with prior research investigating the carbon sequestration capabilities of biochar. Guo and Chen (2014) also observed the substantial capacity of biochar to store carbon and mitigate CO_2_ emissions. Additionally, other studies have emphasized the significant role of pyrolysis temperature in influencing the carbon stability and sequestration potential of biochar, as demonstrated by research conducted by Lehmann et al. (2011) and Liu et al. (2013a, b).

The results of our study have important implications for biochar production and carbon sequestration potential using pigeonpea and maize stalks in India. According to data from the Ministry of Agriculture and Farmers Welfare, under the Government of India (2016–2017), substantial amounts of pigeonpea and maize stalks are produced annually in India (GOI, 2016–17). Based on our findings, we can estimate the potential biochar production, total potential carbon (TPC), and CO_2_ reduction potential for both pigeonpea and maize biochar. Studies were conducted in analysing life cycle assessment of biochar, potential of carbon sequestration and environmental benefits. Fawzy et al. (2022) Life cycle assessment revealed that 2.68 tCO_2_e are embodied per tonne of biochar. This corresponds to a carbon removal capacity of 3.26 tCO_2_ per hour and the removal of approximately 24,450 tCO_2_ annually. Ogawa et al. (2006) has reported highlight diverse applications of biochar for carbon sequestration across different regions. In southern Sumatra, Indonesia, wood residue is transformed into a significant carbon sink, achieving a fixed carbon recovery of 21%. In western Australia, biochar from mallee eucalyptus plantations contributes to salinity prevention and accounts for over half of the total carbon sink in a 35-year project. Meanwhile, in southern Kyushu, Japan, surplus heat from garbage incineration is utilized for carbonizing sawdust, effectively creating a substantial net carbon sink. Yang et al. (2021) conducted a life cycle assessment study to investigate the carbon sequestered and the environmental benefits of biochar. The results demonstrated that converting 1 t of crop residues into biochar could sequester around 920 kg CO_2_ equivalent.

For pigeonpea biochar, our results showed a biochar yield of 34% at 400 °C with 84.60% fixed carbon content. Using this data, we can estimate the biochar production potential, TPC, and CO_2_ reduction potential for pigeonpea biochar in India. The estimated biochar production potential is 6.30 million tonnes per year, while the TPC is estimated to be 5.33 million tonnes per year. Additionally, the CO_2_ reduction potential of pigeonpea biochar is estimated to be in the range of 15.61 million tonnes of carbon dioxide equivalent per year. This suggests that pigeonpea biochar has the capacity to sequester approximately 3.42 million tonnes of carbon annually from agricultural soils.

Similarly, for maize biochar, our study revealed a biochar yield of 29% at 400 °C with 64.85% fixed carbon content. Using these values, we can estimate the biochar production potential, TPC, and CO_2_ reduction potential for maize biochar in India. The estimated biochar production potential is 5.72 million tonnes per year, while the TPC is estimated to be 3.71 million tonnes per year. The CO_2_ reduction potential of maize biochar is estimated to be in the range of 10.86 million tonnes of carbon dioxide equivalent per year. This suggests that maize biochar application in agricultural soils has the potential to sequester approximately 3.42 million tonnes of carbon annually.

These findings indicate the significant carbon sequestration potential of biochar derived from pigeonpea and maize stalks in India. By converting agricultural residues into biochar, a stable form of carbon, we can effectively sequester substantial amounts of carbon dioxide from the atmosphere and mitigate climate change impacts. Implementing biochar application in agricultural practices can contribute to the national carbon sequestration efforts and promote sustainable soil management strategies.

### Energy-related properties

**Fig. 5:**
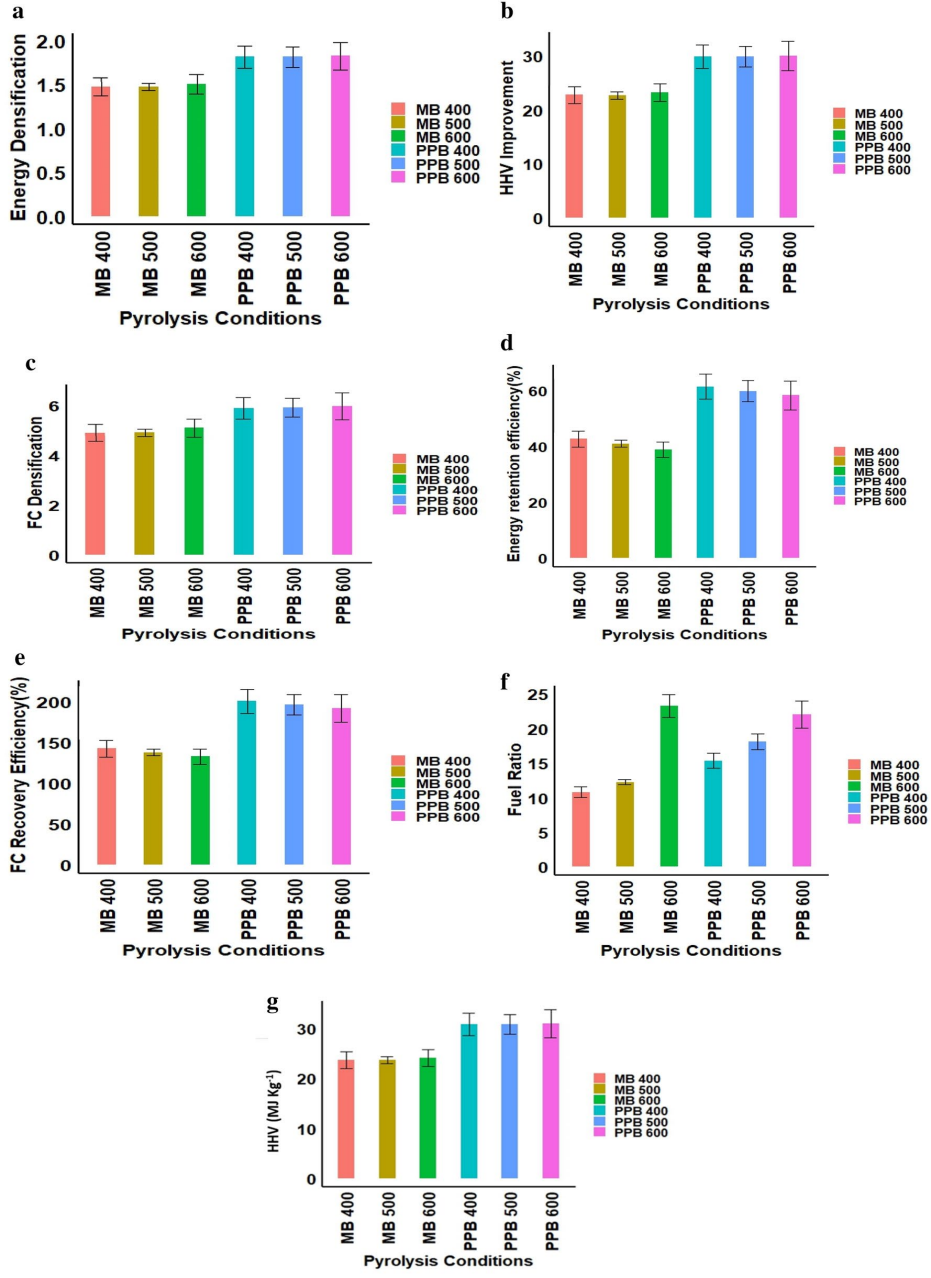
Effect of pyrolysis conditions on energy densification (Ed) (**a**), Energy retention efficiency (**b**), fixed carbon densification (FCd) (**c**), HHV Improvement (**d**), FC recovery efficiency (FCre) (**e**), and fuel ratio (Fr) (**f**), and higher heating value (HHV) (**g**)

Figure 5 showcases estimated values of various energy-related properties of the biochar, including energy retention efficiency, energy densification (Ed), fuel ratio (Fr), higher heating value (HHV), and FC densification (FCd), HHV improvement (HHVi), and FC recovery efficiency (FCre). These factors play a crucial role in evaluating biochar's potential as a solid fuel substitute and energy source (Venkatesh et al 2022a, b). Chemical reactions, such as dehydration and decarboxylation, which release H2O and CO_2_, contribute to improving the energy characteristics of biochar (Libra et al. 2011).

Energy retention efficiency (ERE) is a significant parameter for assessing the efficiency of biochar production as solid fuel substitute. ERE showed a decrease as the temperature increased from 400 °C to 600 °C. Pigeonpea biochar exhibited higher ERE compared to maize biochar. The higher ERE observed in pigeonpea biochar compared to maize biochar (Fig. 5) can be attributed to differences in the chemical composition and structural properties of the two biomasses. At 400 °C, both pigeonpea (61.47–65.94%) and maize (42.66–45.64%) biochar had higher ERE values because of higher biochar yields at that temperature (34% for pigeonpea and 29% for maize). However, ERE decreased at 600 °C for both pigeonpea (58.29–63.51%) and maize (38.89–41.64%) biochar due to reduced biochar yield (29% for pigeonpea and 26% for maize). These results indicate that 400 °C was the optimum temperature for producing energy-rich biochar.

The energy densification values increase as the pyrolysis condition temperature increases, with PPB 600 having the highest energy densification value. PPB 400 and MB 600 have the largest difference in energy densification values, with a difference of 0.337. Pyrolysis condition temperature may have a greater effect on energy densification values than the type of biomass used, with the difference in values between PPB and MB being relatively small. However, the energy densification significantly increases by 0.031 at MB 600 when compared to MB 500. Similar trends in energy densification have been reported for various crop residues (Lu et al 2013; Hoekman et al 2011).

The higher heating value (HHV) is a key factor in determining the fuel value and energy content of biochar. It measures the amount of energy released per unit mass or volume of fuel during complete combustion. It serves as a measure of the fuel’s energy content. It encompasses not only the energy released during combustion, but also the energy carried away by water vaporization (Ghugare et al 2013). HHV values ranged from 30.75 to 32.98 MJ kg^−1^ in pigeonpea biochar and 23.63–25.83 MJ kg^−1^ in maize biochar (Fig. 5). Pigeonpea biochar exhibited a higher HHV at 400 °C, while pigeonpea biochar showed the highest HHV at 500 °C. The variation in HHV of the biochar can be attributed to differences in the ash content (Ronsse et al. 2013). The HHV of pigeonpea biochar produced at 400 °C (30.75–32.98 MJ kg^−1^) slightly exceeded that of dimethyl ether (29 MJ kg^−1^), while the HHV of maize biochar produced at 400 °C (23.72–25.38 MJ kg^−1^) surpassed that of hard black coal (25 MJ kg^−1^) (Valde et al. 2016), indicating their high energy potentials. The increase in higher heating value (HHV) is due to the evaporation of low-energy elements like oxygen, hydrogen, and nitrogen. This results in the enrichment of high-energy carbon content (Yang et al. 2016).

The fixed carbon recovery efficiency decreases as the pyrolysis condition increases, with the highest FCre of 200.45–215.03% at PPB 400 and the lowest FCre of 132.97–142.37% at MB 600. Both pigeonpea and maize biochar displayed higher FCre (200.45–215.03% & 142.37–152.32%), energetic retention efficiency (61.47–65.94% & 42.66–45.64%), fuel ratio (15.38–16.50 & 10.81–11.56%), and HHV (30.75–32.98 MJ kg^−1^). These findings indicate that biochar produced from pigeonpea and maize stalks at 400 °C can serve as an alternative solid fuel (Abdullah et al., 2009).

### Economics of biochar production

In agriculture, particularly in rainfed conditions economics of production plays an important role in the feasibility and applicability of new production technologies. Based on the variables obtained from the kiln runs, an estimation of the cost of producing biochar from the stalks of pigeonpea and maize was performed. The production cost per kilogram of biochar was calculated by considering the data acquired from the conversion efficiency into biochar, kiln operation, and residue load (Table 4). The cost of setting up one kiln was INR 1500/-. One man-day is needed to operate the kiln, while two man-days are required for processing and other operations. For 100 kg of pigeonpea and maize stalk residues, a total of four kiln operational runs were obtained with a conversion efficiency of 28% and 23%, respectively. The average production cost for one kg of biochar was estimated to be INR 9/-. The market price of biochar was assumed to be INR 50/- per kg and with that the BCR (benefit cost ratio) of Pigeonpea was 1.6 and maize 1.3. The results also show that production of biochar from pigeonpea is more economical than the production from maize residue. Keeping the pyrolysis method, kiln operation schedules, residue availability, and conversion ratio constant, the major operational cost is towards labour component. If the model will be introduced to small and marginal farmers, they can use their family labours on a part-time basis and thus the cost towards labour component would be nullified and the net benefits and BCR ratio will be much higher. However, the model is feasible, applicable and more profitable in areas where labour costs are low (Odisha, West Bengal, Bihar, etc.) and surplus. The availability of feed stock is one of the key factors deciding the economics of biochar production. The authors are sceptical about the influence of climate change on renewable energy sources. Osman et al. (2022) emphasize the importance of understanding the effects of climate change on renewable energy production. Their research identifies wind, hydropower, biomass, and geothermal energy as the most impacted by climate change, with solar energy being the least affected.

**Table 4:** Cost of producing biochar from pigeonpea and maize (given in INR: Indian rupees)

Capital investment per kiln	INR per kiln
Cylindrical metal drum	600
Gas cutting of vents	500
Handle fitting and top lid	400
Total cost	1500
Operational cost of biochar production	
Kiln operation (1 man-day)	300
Processing of residues (2 man-days)	600
Cost of production	900
Conversion efficiency of pigeonpea	28%
Conversion efficiency of maize	23%
Cost of production	900
Pigeonpea stalk biochar (kg)	28%
Maize stalk biochar (kg)	23%
Market price of biochar	50%
Gross income- from pigeonpea	1400
Gross income- from maize	1150
BCR-pigeonpea*	1.6
BCR-maize*	1.3

## Conclusions

Through this study, the insights on production of biochar from pigeonpea and maize stalks at different pyrolysis temperatures and their characteristics were obtained. We observed a reduction in the biochar yield from both feedstocks as the pyrolysis temperature increased from 400 °C to 600 °C, declining from 34 to 26%. This decline is influenced by the composition of the feedstock, pyrolysis conditions, and properties of the biomass. Notably, pigeonpea stalk, with its higher lignin content, yielded more biochar than maize stalks. Proximate analysis showed that as pyrolysis temperature increased, volatile matter decreased, and ash content had a slight rise. Pigeonpea biochar, having higher fixed carbon content, displayed better stability and less susceptibility to decomposition than maize-derived biochar.

The analysis of nutrient composition revealed that elevated pyrolysis temperatures led to higher concentrations of vital nutrients, including phosphorus, potassium, calcium, magnesium, sulphur, and various micronutrients, in both pigeonpea and maize biochar. However, nitrogen content decreased with increasing pyrolysis temperature. These results enhance our knowledge of nutrient dynamics in biochar and are consistent with earlier studies. According to Cantrell et al. (2012), biochar produced at lower temperatures is appropriate for regulating fertilizer nutrients and absorbing pollutants from the soil. Higher temperatures lead to material analogous to activated carbon and environmental remediation. The bioavailability of nutrients for example iron and zinc and their effect on plant growth and yield is reported to be influenced by biochar application. This might be due to change in soil pH, improved cation exchange capacity and improved soil microbial activity (Vahedi et al. 2022; Ding et al. 2016). By examining the stability of biochar, we discovered differences in hydrogen and oxygen levels between maize and pigeonpea biochar, which may be due to variations in the materials used to produce them. The pigeonpea biochar had a higher amount of hydrogen and oxygen in comparison to the other biochar. Both biochar was highly carbonized and aromatized, as evidenced by their low hydrogen-to-carbon ratio. As the pyrolysis temperature increased, the oxygen-to-carbon ratio decreased, indicating that oxygen-containing functional groups were removed during the pyrolysis process. Furthermore, this study found that higher pyrolysis temperatures resulted in reduced polycyclic aromatic hydrocarbons in both pigeonpea and maize biochar. The biochar generated during the process at 400 °C pyrolysis temperature has higher biochar yield, carbon fixation (about 84.60% for PPS and 64.85% for maize), energy retention efficiency (61.47–65.94% for PPS and 42.66–45.64% for maize), fixed carbon recovery efficiency (about 200.45–215.03% for PPS and 142.37–152.32% for maize) and HHV (about 30.75–32.98 MJ kg^−1^ for both), implying that it is suitable for use as an alternative solid fuel. Additionally, the biochar characteristics, including nutrient composition and stability, suggest its potential use in soil improvement and carbon sequestration. Notably, there was a significant potential for CO_2_ reduction in pigeonpea and maize biochar. The comprehensive analysis of the results reveals that pigeonpea stalks derived biochar stand superior over maize in terms of biochar yield, energy retention efficiency, fixed carbon content and fuel properties (HHV) which might be due to difference in physiochemical characteristics of maize and pigeonpea stalks. This understanding of biochar characteristics is required for proper usage of biochar as soil amendment, for pollution removal, alternate fuel, etc. However, the usage of maize stalks derived biochar cannot be underestimated as the quality of biochar meets the essential quality criteria for carbon sequestration in soil, as established by the European Biochar Certificate (EBC) and the International Biochar Initiative (IBC). Biochar as soil amendment was reported to improve soil health, and its role in pollutants removal and carbon sequestration is known, however, further long-term in-depth studies are needed to understand the impact of biochar on soil health, environmental sustainability, its effects on soil microbial diversity, and role of biochar in vertical farming as a nutrient source. New avenues of research, vertical farming and its synergy its synergy with hydrogen economy was reviewed in detail by Osman et al. 2023. The study detailed the importance of vertical farming in the context of increasing world population to 9.7 billion by 2050 and the use of biochar in vertical farming systems should be studied in future.

## Future prospects

Further research could focus on a more detailed characterization of biochar properties, such as its chemical composition, surface area, and pore structure. This can help understand how variations in production methods affect biochar quality and performance. It is required to explore the potential of biochar as a soil amendment for specific crops or in specific environmental conditions by conducting long-term field experiments. Different crops and soils may respond differently to biochar, so targeted studies could provide valuable guidance. Understanding how biochar interacts with soil over extended periods can inform sustainable carbon sequestration strategies.

## Data Availability

All data generated or analysed during this study are included in this published article and its supplementary information files.
